# Understanding the Vestibular Apparatus: How 3D Models Can Improve Student Learning

**DOI:** 10.1007/s12070-024-05272-9

**Published:** 2024-12-23

**Authors:** Amanda Ragland, Lauren Linquest, Runhua Shi, Gauri Mankekar

**Affiliations:** 1 LSU Health Shreveport School of Medicine, 1501 Kings Hwy, Shreveport, LA 71103 USA; 2 Department of Medicine, Feist-Weiller Cancer Center, LSU Health Shreveport, 1501 Kings Hwy, Shreveport, LA 71103 USA; 3 Department of Otolaryngology-Head and Neck Surgery, Neurotology, LSU Health Shreveport, 1501 Kings Hwy, Shreveport, LA 71103 USA

**Keywords:** Medical education, Otology, Vestibular dysfunction, Audiovestibular systems, Inner ear disorders

## Abstract

The objective of this study is to compare student satisfaction and confidence following a presentation of the inner ear anatomy using either a 2D model or a 3D model. First-year medical students were randomized to a 2D or 3D teaching group and given a presentation on the inner ear. Students were surveyed on confidence levels pre- and post-presentation, helpfulness of the presentation, and completed the student satisfaction and self confidence in learning (SCLS) questionnaire. 30 first-year medical students participated in the study: 14 in the 2D model and 16 in the 3D model group. A statistical significance was found between the 2D and 3D group regarding the helpfulness of the model (*p* = 0.0147), Q1 of the SCLS questionnaire (*p* = 0.0365), and Q12 of the SCLS questionnaire (*p* = 0.0308). The use of a 3D model of the inner ear aids in student self-confidence regarding the material and is perceived to be helpful with learning the material.

## Introduction

Medicine is an ever-changing field with new discoveries and advancements that medical education must keep up with [1]. Traditional medical education was centered on textbook learning and chalkboard lectures. While these two-dimensional (2D) teaching methods have been successful for many generations of medical students, advancements in technology offer new ways to enhance the learning experience [2].

Three-dimensional (3D) printing originated in the 1980s and has expanded into a multitude of fields, including medicine [3]. Many studies have shown the benefits of 3D models, especially their ability to be reproducible and have a high level of correctness when conducting research on a particular topic [4]. 3D education encourages critical thinking via active learning, offering a hands-on experience. A study performed by Bui et al. demonstrated the superior learning outcomes that 3D models offer with students exhibiting improved long term retention of hard-to-grasp concepts compared to a 2D model group [2].

Anatomy is core to medical education [5]. Imaging interpretation, including computed tomography (CT) and magnetic resonance imaging (MRI), relies heavily on anatomical knowledge and spatial relationships. This is a common struggle among medical students when first learning how to read these images [2]. In addition to imaging, surgical interventions require a mastery of anatomy that 2D platforms may not fully offer. Cadaver dissection has been the traditional means of anatomical education but has limitations including cost, delivery, and maintenance [2, 4]. This is why studies such as one performed by Youman et al. (2021) have suggested the use of 3D models as a new form of teaching method in medical education [3].

Teaching via 3D models allows better visualization and perception with an enhanced understanding of anatomy. The models are created to offer a concrete structural entity that correlates with disease and anatomy that other educational platforms cannot achieve [6]. Regarding imaging education, 3D models can transform CT and MRI images into solid and graspable objects with a multidirectional view to enhance student understanding [2]. Surgically, 3D anatomical models can be used for procedural preparation in a way that 2D platforms cannot [3]. Anatomical knowledge can be especially difficult to grasp for complex and intricate areas of the body, including the inner ear. Because this domain is extremely small and concealed, it is often perplexing to medical students, especially via a 2D platform. Regardless, the inner ear anatomy is crucial to comprehend before understanding the disease states associated with this area [7].

The goal of this study is to consider the role of a 3D vestibular model in medical student education of the inner ear. This study aims to achieve this goal by:

- Providing an identical teaching activity to two study populations, apart from the model used.
- Comparing questionnaire responses regarding confidence of material pre- and post-demonstration, as well as helpfulness and satisfaction of the activity.
- Analyzing the differences in questionnaire responses between the two groups.
- Determining if medical students subjectively report a difference in the experience of learning inner ear anatomy with a 2D model versus a 3D model.
- Using this comparison to consider the benefit of adding 3D models to medical education for complex anatomical fields, such as the inner ear.

By comparing a 2D versus a 3D model of the vestibular system, this study hopes to further understand how 3D models can change students' outlooks on their understanding of inner ear anatomy and physiology.

## Materials and Methods

### Population

This study was conducted at LSU-Health Shreveport medical school. Inclusion criteria included any medical student currently enrolled in the medical school class of 2026. Exclusion criteria were any other medical student not enrolled in that medical school class. The class of 2026 students were emailed one week prior to the teaching session and asked to voluntarily participate in the study. No compensation or other insinuating factors were offered for participation.

### Teaching Activity

**Fig. 1:**
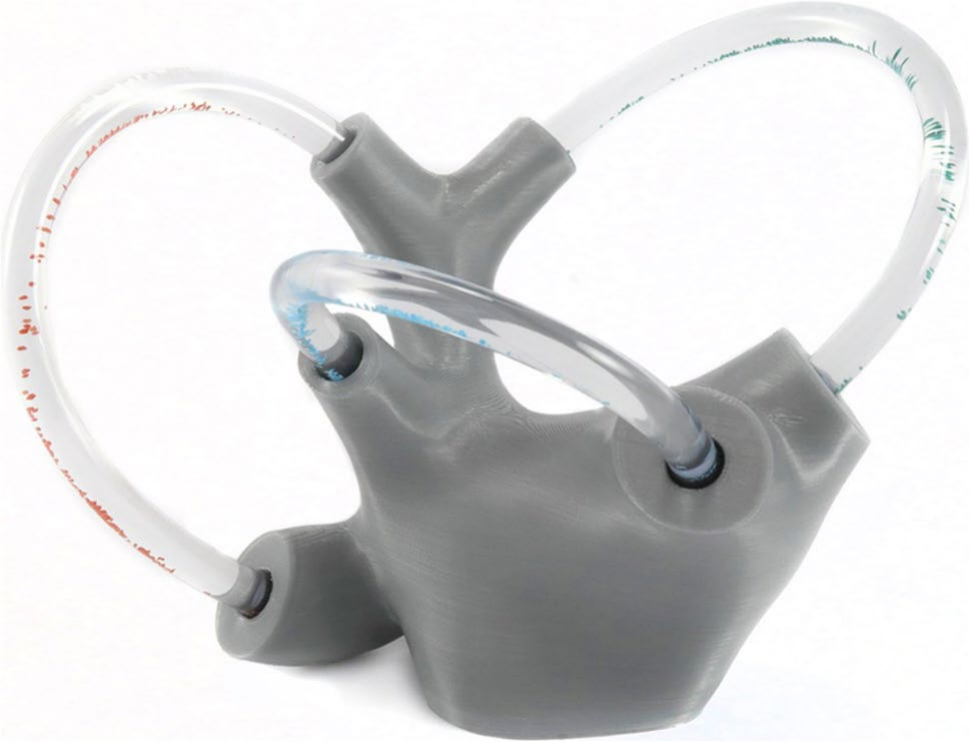
2D model of the inner ear and vestibular apparatus, provided by Vestibular First ©

**Fig. 2:**
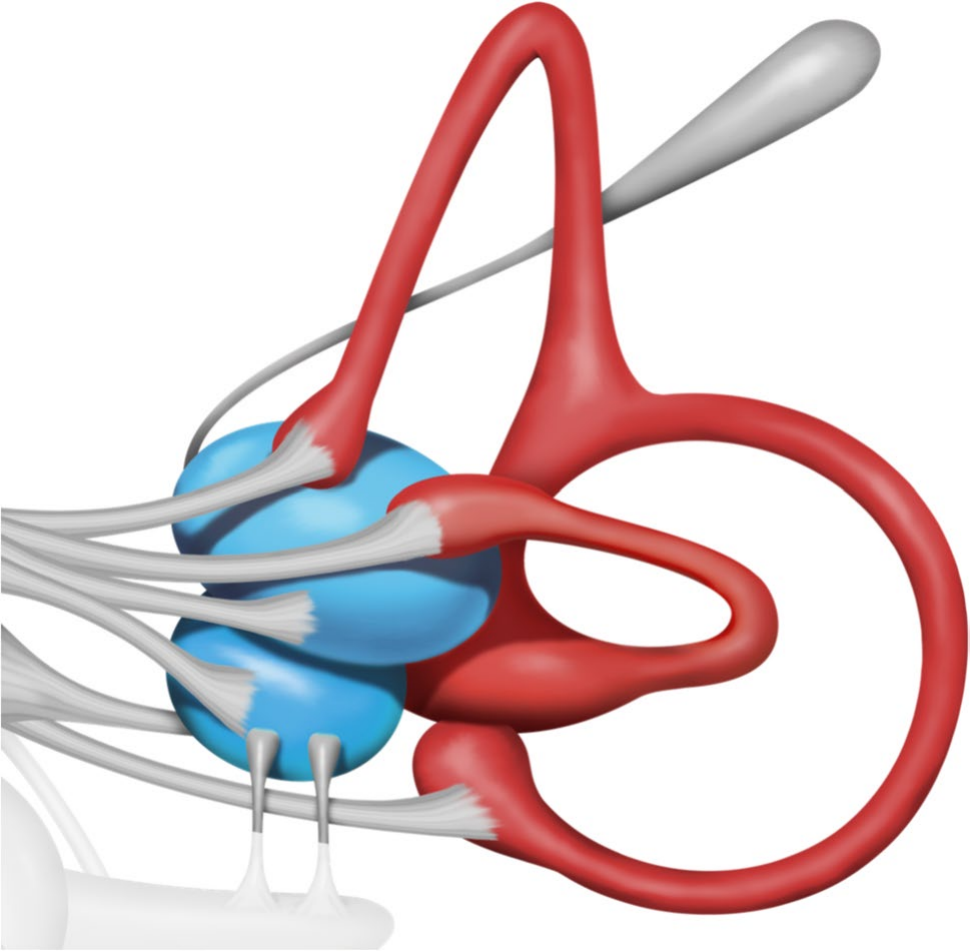
3D model of the inner ear and vestibular apparatus, provided by Vestibular First ©

Individuals who decided to participate were randomly assigned to one of two groups. One group was to have a pre-teaching session using a 2D image of the vestibular apparatus, as shown in Fig. 1. The second group would have a pre-teaching session using a 3D model of the vestibular apparatus, as shown in Fig. 2. In this teaching session, a broad overview of how the vestibular apparatus operates and how this system senses changes in position were explained. A script was written to ensure the same information was presented in each group. However, the groups differed by the model of the inner ear that was used to explain the information. In the 3D model group, participants were able to hold the model while the teaching session was being performed. The 2D group had an image of the model to be referenced during the teaching session. Both the 3D model and 2D images were provided by Vestibular First. Vestibular First provided no financial compensation or other funding for this project. Following the teaching session, all students from the 3D and 2D groups attended the same lecture given on the pathology of the inner ear related to the vestibular apparatus.

### Data Collection

**Fig. 3:**
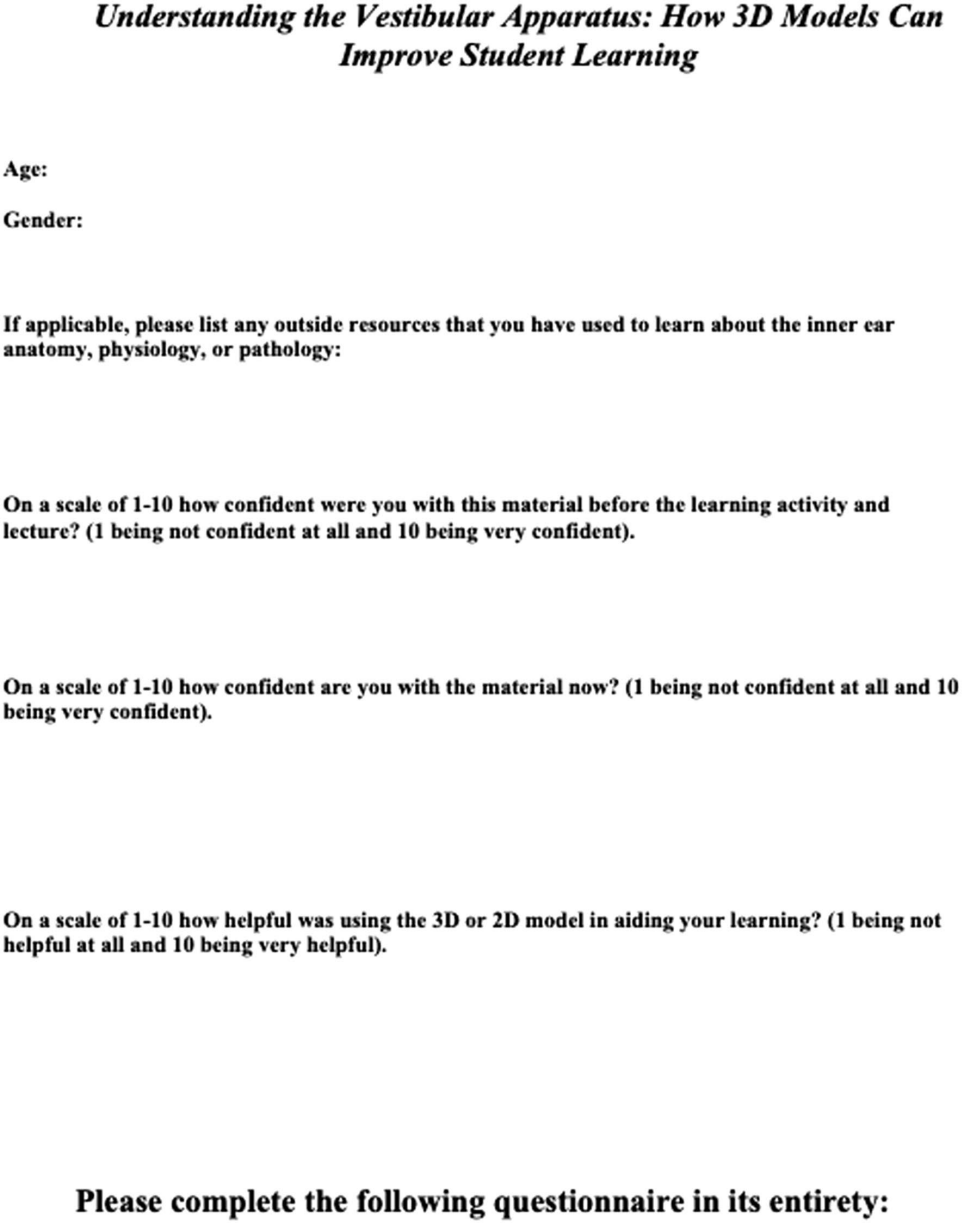

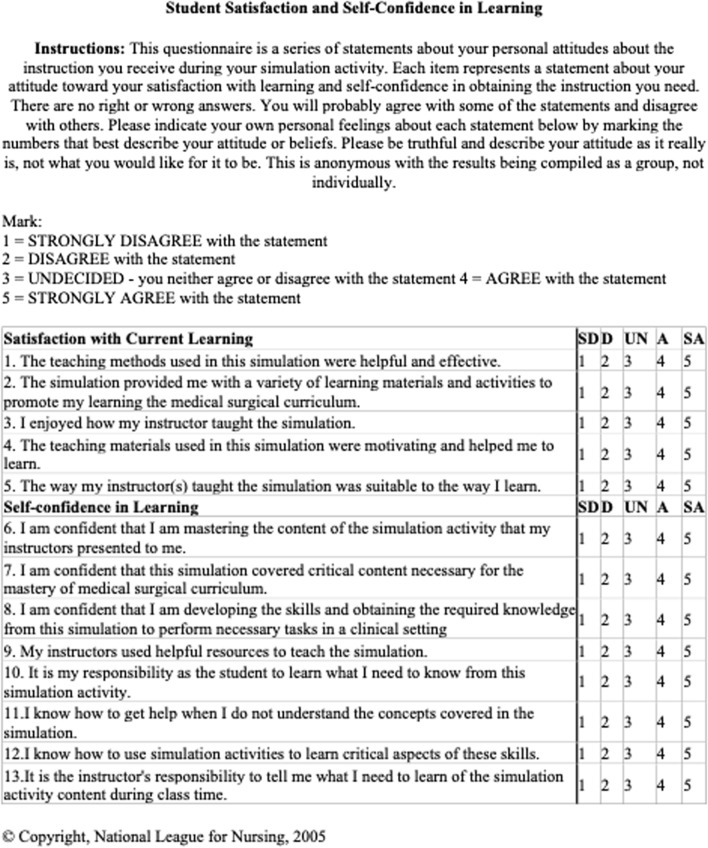
Student questionnaire including SCLS survey

Following the teaching activity and lecture, students were asked to fill out a questionnaire. Data collected included gender, age, and previous outside learning resources used to study the inner ear. Students were then asked to rate on a scale of 1–10 (10 being extremely confident) how confident they felt about the material before the teaching activity. They were then asked to rate on a scale of 1–10 how confident they were about this material following the teaching activity. Additionally, students quantified how helpful the 2D or 3D learning activity was on a scale of 1–10 (10 being extremely helpful). Lastly, participants filled out the Student Satisfaction and Self Confidence in Learning (SCLS) questionnaire [8]. This is a validated and standardized questionnaire to assess students’ feelings towards a particular simulation activity, in this case to regarding the pre-teaching session. A Likert scale of 1–5 is used with 1 strongly disagreeing and 5 strongly agreeing with the statement. All information was gathered anonymously and separated by 2D vs 3D group. A copy of the survey and SCLS questionnaire are found in Fig. 3.

### Statistical Analysis

A biostatistician on campus, Dr. Shi, was consulted, and a two-group study design was suggested as follows: A sample size of 27 in each group will have a 95% power to detect a difference in means of − 15 (the difference between a mean, µ_1_, of 193 and a mean, µ_2_, of 208) assuming that the common standard deviation is 15 using a two-group *t* test with a 5% two-sided significance level. Descriptive statistics such as means and standard deviations were calculated for each question and subject measurement in the SCLS survey. The difference between the student responses to the questions pertaining to confidence before and after the teaching session was computed for the 2D and 3D model groups. According to the SCLS questionnaire, overall satisfaction can be computed from questions 1–5. The helpfulness of the teaching session was also measured. Chi-square and student t-tests were performed when appropriate on respective analyses. All statistical analysis was performed by using the Statistical Software SAS 9.4 for Windows [9]. All *p* values < 0.05 were considered statistically significant.

## Results

A total of 30 students decided to participate in this study. While individuals were randomized to the 2D or 3D groups, one participant accidentally participated in the wrong group. Thus, 14 individuals participated in the 2D teaching session and 16 individuals participated in the 3D learning session. An overview of the study population can be found in Table 1. Among the 2D model group participants, 64.29% were female and in the 3D model group, 62.5% were female. No statistically significant difference in gender was found between the two groups (*p* = 0.9193). The average age of the cohort was 23.7 years old (SD 1.67 years) and there was no statistically significant difference between the two groups (Tables 2 and 3, *p* = 0.7874).

**Table 1:** Characteristics of all participants

Levels	N	Percent
*Model*		
2D	14	46.67
3D	16	53.33
*Gender*		
F	19	63.33
M	11	36.67
*Age*		
Mean	23.77	
Std Dev	1.68	
Total N	30

**Table 2:** Survey statistics for the 2D vs 3D model groups

Question	2D Model (N = 14)				3D Model (N = 16)			
	Mean	Median	Std Dev	N	Mean	Median	Std Dev	N
Age	23.86	23	1.92	14	23.69	23.5	1.49	16
Confidence Pre	5.00	5.5	1.80	14	5.06	5.5	1.88	16
Confidence Post	7.21	8	1.37	14	7.81	8	0.83	16
Helpful	5.92	6	2.56	13	8.00	8	1.71	16
Q1:	3.86	4	0.86	14	4.31	4	0.48	16
Q2:	3.21	3	0.97	14	3.75	4	0.93	16
Q3:	3.93	4	0.92	14	4.31	4	0.79	16
Q4:	3.57	3.5	1.02	14	4.06	4	1.06	16
Q5:	3.93	4	1.21	14	4.25	4	0.68	16
Q6:	3.50	3.5	0.94	14	3.75	4	0.68	16
Q7:	3.93	4	1.00	14	4.13	4	0.72	16
Q8:	3.57	4	1.09	14	3.94	4	0.85	16
Q9:	3.93	4	0.73	14	4.25	4	0.58	16
Q10:	4.43	5	0.76	14	4.31	4.5	0.87	16
Q11:	4.07	4	0.83	14	4.38	4.5	0.81	16
Q12:	3.07	3	1.07	14	4.06	4	0.68	16
Q13:	4.00	4	0.88	14	3.94	4	1.00	16

**Table 3:** Comparison measurements between the 2D vs 3D model groups

Variable	2D Model (N = 14)			3D Model (N = 16)			*p* value*
	Mean	Median	Std Dev	Mean	Median	Std Dev	
Age	23.86	23	1.92	23.69	23.5	1.49	0.7874
Difference Confidence (Post–pre)	2.21	2	1.53	2.75	2	1.61	0.3603
Helpful	5.92	6	2.56	8.00	8	1.71	0.0147

*Student *t* test used

Online resources and previous lectures on inner ear anatomy were the most reported pre-learning resources used to the material before the teaching session. Seven individuals did not report using any outside resources prior to the activity. There was no statistically significant difference between the groups on prior learning of this material. The mean confidence score prior to the teaching activity in the 2D group was 5 (SD 1.80) and in the 3D group was 5.06 (SD 1.88). The confidence following the teaching session for the 2D group was 7.21 (SD 1.37) and the 3D group was 7.81 (SD 0.83) (Table 2). There was no statistically significant difference found between the 2D or 3D groups based on confidence before or after the teaching session. However, a significant difference was found between the 2D teaching session group (5.9 ± 2.56) and the 3D session group (8 ± 1.71) in helpfulness rating (*p* = 0.0147) (Table 2).

The mean and standard deviation of the responses to the questions of the SCLS survey can also be found in Table 2. A significant difference was detected between the 2D (4 ± 0) and 3D groups (4.33 ± 1.75) in responses to question 1 which related to if the teaching session information was helpful and effective (*p* = 0.0365). Additionally, a significant difference was detected between the groups for question 12 (2D 3.07 ± 1.07, 3D 4.06 ± 0.68) which asked students to rate their self-confidence in their ability to use the activity to learn skills (*p* = 0.0308). No significant difference was found in responses to the other questions in the SCLS survey.

## Discussion

The medical school curriculum seems to be ever-changing with advances in technology and educational innovations becoming extremely beneficial in optimizing student learning. 3D models are the perfect example of an opportunity to give students a more in-depth and personal learning experience. The Vestibular First 3D model of the inner ear used in this study provides support for the utilization of anatomical models to provide a better learning experience as compared to 2D learning via PowerPoint.

The transition from textbooks and lectures into active learning seems daunting, yet it is a necessary adjustment in medical education. In the hands-on world of medicine, medical students need more opportunities for immersive experiences to supplement classroom learning. There are numerous drawbacks of passive learning, including inefficient use of time, lack of flexibility in a busy schedule, and inability to accommodate all learning styles [10]. In addition, lectures lack an opportunity for critical thinking, and in subjects such as anatomy, critical thinking is vital to comprehend spatial relationships [11]. Medical knowledge is already challenging to acquire and retain, and lectures do not address this properly [12]. Medical professionals are expected to be lifelong learners, therefore providing medical students with an immersive learning opportunity beyond the lecture hall lays a foundation for successful sustained retention of knowledge. Active learning allows students to go beyond memorization and obtain an immersive learning experience. Student performance and inclusivity increase with active learning, as the concepts can be recalled outside of a classroom setting, and a more complete foundation of the subject matter is established [13]. At its core, implementing active learning relies on learner participation [14]. The addition of 3D models in subjects such as anatomy is the perfect example of emphasizing learner participation, as it gives students the opportunity to see and feel spatial relationships.

3D models have already been implemented in a variety of curriculums and medical programs. A study by Tripodi, et al. (2020) explored the use of 3D-printed upper limb bones among first-year osteopathic students at Victoria University. The bones were used in a variety of activities such as identifying orientation and landmarks as well as forming muscles with adhesive to overlay on the bones and peer-to-peer presentations. The results of using 3D-printed bones were overwhelmingly positive, with an increase in performance and a reportedly positive experience with students reporting increased confidence and reduced anxiety with learning this subject matter [15]. Another study completed by Li et al. (2015) at Binzhou Medical College compared three groups of students learning spinal fractures: one group was given CT images, one group was given images of a 3D model, and one group was given a 3D printed spinal fracture model. The results of the post-assessment demonstrated that the 3D-printed model group had improved identification of the spinal fracture anatomy [16]. Veterinarian students have also proven that 3D models improve performance in anatomy, as illustrated by Preece et al. (2013). Third-year veterinary medicine students were separated into three learning groups, one being a physical model of an equine foot, another using a textbook model of an equine foot, and a third using a 3D computer model of an equine foot. Each group was tested on anatomical identification on an MRI scan after learning with their assigned model. The students who used the physical model had higher assessment scores and reported more positive feedback than the other two groups [17]. These three studies demonstrate the wide application and benefit of 3D models especially with anatomical learning.

Although this study does not evaluate student performance, it does evaluate student perception of the use of 3D models when learning inner ear anatomy. Student perception is vital to consider when implementing active learning models into the medical curriculum. In this study, a significant difference was found in the individual helpfulness rating between the 2D model group and the 3D model group. Helpfulness was also assessed in question 1 of the SCLS questionnaire which related to helpfulness and effectiveness of the teaching session information. Together, this further supports the perceived helpfulness of the 3D model in learning inner ear anatomy. In addition to helpfulness, this study found a significant difference in student-perceived self-confidence in the ability to use this activity to learn skills, indicated by question 12 of the SCLS questionnaire. This implies that 3D model group felt more confident in learning skills, which supports the use of 3D models in learning complex anatomy. These results are in accordance with the student commentary of the study conducted by Tripodi, et al. (2020) regarding increased confidence and effectiveness when using a 3D model of anatomical matter [15].

Question 12 of the SCLS questionnaire in which students of the 3D model indicated a higher level of confidence in learning skills emphasizes an important aspect of active learning. Active student learning, including the use of 3D models, helps with applied skills that will be used while in practice. Specifically, 3D anatomical models can help in procedural fields such as surgery when preparing for operations as this field heavily relies on spatial awareness. Ghazi, et al. (2020) analyzed the role of 3D printed models in urology laparoscopic surgical education and emphasized the need for 3D model simulations due to the ability of the learners to practice their psychomotor and judgment skills in an environment where no harm could occur to patients [18]. Similarly, simulation labs with mannequins allow students to apply the learned skills in a controlled environment which increases confidence and capability in these required skills. Second-year medical student skills were evaluated in a study by Yamazaki et al. (2020) regarding blood pressure measurement. The simulation-based practice of taking a mannequin’s blood pressure was compared to traditional training, and the students using simulators had improved measurement skills, retained those skills, and illustrated proficiency in clinical practice [19]. Active learning in the format of 3D printed models has the potential for students to retain skills and proficiency in clinical settings that require comprehensive knowledge of anatomy.

Limitations that were encountered in this study included a small sample size of only 30 participants out of the 150 first-year medical students at the institution. We recognize that a total of 30 participants may not allow us to see a meaning difference in our current study. It may indicate that the 2D model may have different results if we had enough participants recruited in our study. A smaller sample size indicates that the results may not reflect the perceptions of most students in the class. Selection bias is present due to the small sample size of only 30 medical students. The intended population is medical students actively learning inner ear anatomy, and this study’s small population cannot represent all medical students nor their subjective opinions of the effectiveness and helpfulness of the anatomical models. Another limitation included using the SCLS questionnaire which was produced by the National League for Nursing, and the questions were simulation-based, not 3D model-based, which was not as specific for this study. Although it was able to evaluate active learning skills, a survey that is based on 3D-model education would have been more specific to this study. Student perception was successfully measured, but an additional limitation is that academic improvement was not measured in this study, which lacks support for 3D model use for greater academic success. Due to the nature of the study, there were no objective measures obtained to demonstrate that students achieved a better understanding with the 3D model; the only goal of the study was to obtain subjective opinions from the students. This opens the opportunity for a future study to be conducted that investigates objective academic success by measuring retention of information with a 2D model versus a 3D model. Further limitations include the methodology of the 2D and 3D presentations, as each presenter was a different student, which leads to human error in presentation efficacy despite the script that each presenter followed being identical. The limitation of having these uncontrolled variables, while not being totally avoidable, can be addressed if the same presenter had given each presentation in the same environment to remove as much bias as possible.

## Conclusion

After 2D and 3D model presentations of the inner ear anatomy, this study found that students who received a 3D model presentation felt more confident in their understanding of the material and felt that the presentation was more helpful than the 2D model participants. Due to the inability to measure assessment success about the material, this study cannot confidently report that 3D model use leads to academic success. However, this study can report that students perceive that 3D models can help with learning this complex anatomical material. The limitations of this study, including the lack of objective outcomes, provides the opportunity for future studies. A larger sample size can be included in a follow-up study to remove selection bias from the outcomes. In addition, a questionnaire that uses different verbiage could be included to ensure the participants are considering the model instead of a simulation, and the same instructor would be present for both teaching sessions. Lastly, objective performance could be measured if a validated assessment were used following the teaching lessons, which could possibly strengthen the support for the use of 3D models in anatomical education. Although objective measures were not obtained, student perception is still vital to recognize when making changes to the medical education curriculum, and this study shows support that students may positively accept active learning.

For readers who are interested in obtaining 2D and 3D models, visit vestibularfirst.com for purchasing information, as well as guides with DIY instructions for printing a personal vestibular apparatus.
